# Synthetic magnetic resonance-based relaxometry and brain volume: cutoff values for predicting neurocognitive outcomes in very preterm infants

**DOI:** 10.1007/s00247-024-05981-x

**Published:** 2024-07-09

**Authors:** Tim Vanderhasselt, Maarten Naeyaert, Nico Buls, Gert-Jan Allemeersch, Steven Raeymaeckers, Hubert Raeymaekers, Nathalie Smeets, Filip Cools, Johan de Mey, Jeroen Dudink

**Affiliations:** 1 Department of Radiology, Vrije Universiteit Brussel, Universitair Ziekenhuis Brussel, Laarbeeklaan 101, 1090 Brussels, Belgium; 2 Department of Pediatric Neurology, Vrije Universiteit Brussel, Universitair Ziekenhuis Brussel, Brussels, Belgium; 3 Department of Neonatology, Vrije Universiteit Brussel, Universitair Ziekenhuis Brussel, Brussels, Belgium; 4 Department of Neonatology, Wilhelmina Children’s Hospital, University Medical Center Utrecht, Utrecht, the Netherlands

**Keywords:** Biomarkers, Brain, Infant, Newborn, Premature, Synthetic magnetic resonance imaging

## Abstract

**Background:**

Early neurorehabilitation can enhance neurocognitive outcomes in very preterm infants (<32 weeks), and conventional magnetic resonance imaging (MRI) is commonly used to assess neonatal brain injury; however, the predictive value for neurodevelopmental delay is limited. Timely predictive quantitative biomarkers are needed to improve early identification and management of infants at risk of neurodevelopmental delay.

**Objective:**

To evaluate the potential of quantitative synthetic MRI measurements at term-equivalent age as predictive biomarkers of neurodevelopmental impairment and establish practical cutoff values to guide clinical decision-making.

**Materials and methods:**

This retrospective study included 93 very preterm infants who underwent synthetic MRI at term-equivalent age between January 2017 and September 2020. Clinical outcomes were assessed using the Bayley-III scale of infant development (mean age 2.1 years). The predictive value for impaired development was analyzed using receiver operating characteristic curves for synthetic MRI-based volumetry and T1 and T2 relaxation measurements.

**Results:**

The T1 relaxation time in the posterior limb of the internal capsule was a potent predictor of severe (sensitivity, 92%; specificity, 80%; area under the curve (AUC), 0.91) and mild or severe (AUC, 0.75) developmental impairment. T2 relaxation time in the posterior limb of the internal capsule was a significant predictor of severe impairment (AUC, 0.76), whereas the brain parenchymal volume was a significant predictor of severe (AUC, 0.72) and mild or severe impairment (AUC, 0.71) outperforming the reported qualitative MRI scores (AUC, 0.66).

**Conclusion:**

The proposed cutoff values for T1 relaxation time in the posterior limb of the internal capsule and for total brain volume measurements, derived from synthetic MRI, show promise as predictors of both mild and severe neurodevelopmental impairment in very preterm infants.

**Graphical Abstract:**

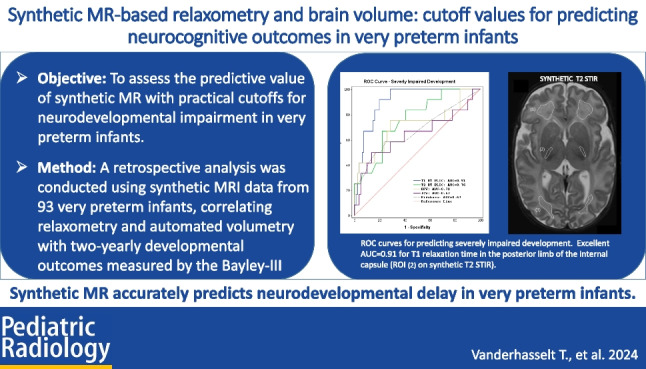

**Supplementary Information:**

The online version contains supplementary material available at 10.1007/s00247-024-05981-x.

## Introduction

Despite recent advancements in neonatal care and a marked decrease in the incidence of severe white matter injuries, very preterm infants, born before 32 weeks of gestation, continue to face a high risk of neurodevelopmental delay [1, 2]. Identifying children who might benefit from early neurorehabilitation is critical for improving their neurocognitive outcomes, particularly during the early years of elevated neuroplasticity [3–5]. However, conventional magnetic resonance imaging (MRI) fails to detect subtle microstructural changes, and its prognostic value, when used as a sole predictor, remains limited [6–9]. Consequently, there is a pressing need for early quantitative biomarkers to predict neurodevelopmental outcomes in daily clinical practice, enabling the selection of candidates for early neurorehabilitation [4].

Brain volumetry represents a promising biomarker that could serve this purpose. Reductions in brain volume at term-equivalent age have been consistently associated with later neurodevelopmental delay [10–12]. However, the technical complexity of neonatal brain MRI volumetry confines its application to specialized research centers, limiting its widespread use in routine clinical imaging [6, 13].

Another promising biomarker under investigation is MRI relaxometry. Indeed, quantitative T1 (longitudinal) and T2 (transversal) relaxation time measurements offer valuable insights into early brain maturation and myelination, processes characterized by decreasing relaxation times [14–17]. However, MRI relaxometry has traditionally been time-consuming. Consequently, studies on MRI relaxation times at term-equivalent ages in very preterm children are scarce, and a correlation with later neurodevelopment has not yet been thoroughly established [14, 16, 18–20].

Synthetic MRI holds considerable promise in the field of neuroimaging [21]. This Food and Drug Administration (FDA)-approved and commercially available method generates quantitative T1, T2, and proton density maps from a single 6-min multi-delay multi-echo MRI sequence. This quantitative data facilitates both neonatal brain volumetry and relaxometry, making it suitable for everyday clinical use beyond research center environments. A key advantage of synthetic MRI is its ability to generate high-quality images from these acquired quantitative maps, comparable to conventional MRI, and thus obviating the need for extended scan times while simultaneously gathering quantitative volumetric and relaxometry data [21–25].

A previous study found that, compared to those with an uncomplicated course, children who experienced severe postnatal morbidity during their stay in the neonatal intensive care unit exhibited prolonged T1 and T2 relaxation times. Notably, the most pronounced prolongation of relaxation times was observed in the posterior limb of the internal capsule [26]. Additionally, two recent feasibility studies have investigated relaxometry in high-risk preterm infants and infants born up to near-term (<37 weeks) with histories of hypoxia or seizures. Both studies found prolonged white matter relaxation times in association with poor neurodevelopmental outcomes at the age of 18–24 months [27, 28]. These findings suggest that MRI relaxometry might serve as a viable biomarker for neonatal brain development.

However, whether prolonged relaxation times at term-equivalent age in non-selected very preterm infants, born before 32 weeks, can serve as prognostic indicators for later neurodevelopment remains unclear.

We therefore aimed to investigate the relationship between synthetic MRI-based relaxation values and brain volume readings in very preterm infants, taken at a time equivalent to full-term age, and their future motor and cognitive capabilities at the age of 2 years. We hypothesized that these synthetic MRI-based measurements could predict developmental delay and define useful cutoff values to assist in clinical judgment and early intervention.

## Materials and methods

### Patients

This retrospective study was conducted by the Department of Radiology and Neonatology at the University Hospital Brussels and approved by the local ethics committee (EC-2022-396). The requirement for informed consent was waived due to the retrospective nature of the study.

We included 140 very preterm (<32 weeks) infants who underwent a synthetic multi-delay multi-echo MRI sequence as part of their routine MRI protocol between January 2017 and September 2020. Our institution routinely conducts MRI scans at term-equivalent age for all very preterm infants, resulting in an unselected cohort. Six patients were excluded from the analysis because of excessive motion artifacts. Clinical outcome data were available for 93 infants and were assessed using the Bayley-III Scales of Infant Development [29] at a mean age of 2.1 years (range, 1.0–3.0 years; standard deviation, 0.44 years).

**Fig. 1:**
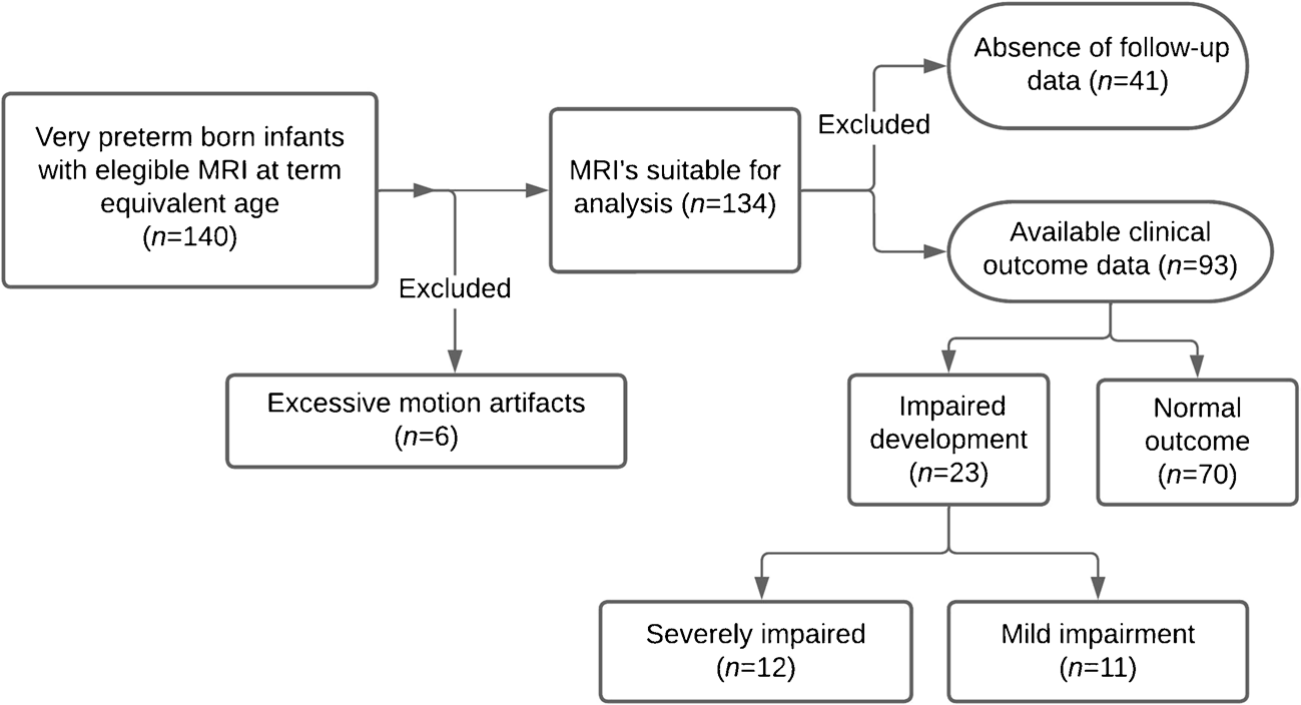
Study flow diagram. *MRI* magnetic resonance imaging

Within this cohort, 23 patients exhibited impaired development, defined as a Bayley-III score of <80 or a confirmed clinical diagnosis of cerebral palsy. Of these 23 patients, 11 had mild impairment (Bayley-III score of 70–79), and 12 had severe impairment (Bayley-III score <70 or cerebral palsy). Figure 1 presents an overview of the data flow.

In previous research, we reported on the diagnostic accuracy of synthetic MRI and the reliability of volumetric analysis for 110 of the 140 patients [23]. We also reported prolonged relaxation times at term-equivalent age in preterm-born children with significant postnatal morbidity for 70 of the 140 patients [26]. The present study addresses the predictive value of synthetic MRI measurements for long-term neurocognitive outcomes after 2 years.

Clinical data were obtained from the hospital electronic records and pseudonymized prior to analysis.

### Magnetic resonance imaging

All children were scanned on a 3-tesla (T) Ingenia MRI scanner (Philips Medical Systems, Best, The Netherlands) with a 16-channel head coil. Patients were sedated with hospital pharmacy compounded solution of chloral hydrate (25–50 mg/kg) and stabilized in a MedVac vacuum immobilization system (CFI Medical Solutions Inc., Fenton, MI). Neonatal earmuffs (MiniMuffs; Natus Medical, San Carlos, CA) and headphones (Würth Group, Künzelsau, Germany) provided appropriate ear protection.

To quantitatively map T1 and T2 relaxation times using synthetic MRI, we utilized a multi-delay, multi-echo acquisition that covered the entire brain. This sequence had a field of view (FOV) of 181 mm and a voxel size of 0.7 × 0.9 × 3.0 mm, with a duration of 6 min. Including the time for conventional 3-dimensional (D) T1 fast spoiled gradient echo, 3-D T2 turbo spin echo, and diffusion-weighted, susceptibility-weighted, and multishell diffusion tensor imaging sequences, the total scanning time was approximately 31 min. The details of the imaging parameters are provided in Online Supplementary Material 1. Quantitative T1 and T2 relaxation maps and fully automated volume measurements were calculated in less than 1 min using SyMRI software version 11.1 (SyntheticMR AB, Linköping, Sweden) integrated into the hospital’s radiology Picture Archiving and Communication System (PACS) system. The automated volume measurements included total brain parenchymal volume, intracranial volume, cerebrospinal fluid volume, and brain parenchymal fraction, defined as the proportion of the brain parenchymal volume to the intracranial volume.

**Fig. 2:**
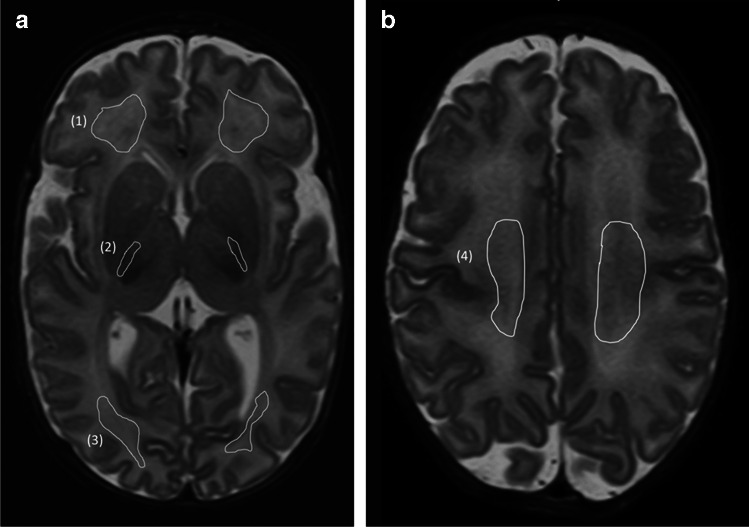
Representative axial synthetic reconstructed T2 short tau inversion recovery magnetic resonance images at the level of the basal ganglia (**a**) and the centrum semi-ovalae (**b**) demonstrate manually delineated regions of interest (ROIs) in a girl scanned at a postmenstrual age of 40 weeks. There is abnormally prolonged T1 (1582 ms) and T2 (141 ms) relaxation times in the posterior limb of the internal capsule above the defined cutoff thresholds, indicating impaired development. This girl exhibited impaired development at 2 years, despite conventional MRI showing no abnormalities. *1* frontal white matter; *2* posterior limb of the internal capsule; *3* parietal white matter; *4* central white matter of the centrum semiovale

Manual regions of interest (ROIs) were outlined by an experienced pediatric neuroradiologist (T.V.), who has 15 years of experience in neonatal MRI, blinded to the study outcomes, on synthetic T2 short tau inversion recovery images as previously described [26]. These ROIs were delineated in the frontal and parietal white matter and the posterior limb of the internal capsule at the level where the basal ganglia were displayed most prominently. A ROI was also placed on the central white matter of the centrum semiovale, on a slice just above the lateral ventricles. Any white matter lesions and cysts were meticulously excluded from the ROI (Fig. 2). The SyMRI software automatically provided the mean T1 and T2 relaxation time values.

### Statistical analysis

All statistical analyses were performed using SPSS version 28 (IBM Corp., Armonk, NY).

The children were categorized into two groups: normal outcome and impaired development. Differences in clinical characteristics between the two groups were assessed using Welch’s *t*-test for continuous variables and the chi-square test for categorical variables. *P*-values <0.05 were considered significant. Correlations between Bayley-III motor and cognition scores, relaxation times, and brain volume were examined using Pearson’s correlation. Welch’s *t*-test was used to evaluate the differences in mean relaxation times between outcome groups and to examine potential differences in mean volume measurements derived from synthetic MRI. Bonferroni correction with a threshold of *P<*0.004 was applied to determine the significance threshold, adjusting for multiple comparisons.

The relationship between MRI measurements and postmenstrual age at the MRI scan was assessed using simple linear regression.

A receiver operating characteristic (ROC) curve analysis was conducted to determine whether relaxation values and brain volume measurements could serve as predictive biomarkers of impaired development. Youden’s index was utilized to determine the cutoff values for sensitivity and specificity. Stepwise logistic regression was used to determine whether a combination of predictive biomarkers could provide an increased area under the curve (AUC).

## Results

### Study patients

Table 1 presents the demographic characteristics of the normal outcome (*n*=70) and impaired development (*n=*23) groups, encompassing known risk factors for developmental impairment [1] and the Kidokoro MRI score of global brain abnormalities provided in the radiological report for all patients. This MRI score is routinely used to report brain abnormalities in preterm infants at term-equivalent ages in a standardized manner [30].

**Table 1:** Demographic characteristics

	Normal outcome (*n*=70/93)	Impaired development (*n*=23/93)	*P-*value^a^
Gestational age (weeks) at birth^b^	29.3 (2.0)	28.1 (2.8)	**0.03**
Postmenstrual age at scan (weeks)^b^	39.8 (0.8)	40.0 (0.8)	0.25
Birth weight (g)^b^	1314 (361)	1094 (425)	**0.02**
Skull circumference at birth (cm)^b^	27.4 (2.9)	25.9 (3.2)	0.05
Days ventilated^b^	2.3 (8.7)	13.4 (20)	**<0.001**
2^nd^ APGAR^b^	8.7 (1.1)	7.9 (1.4)	**0.01**
Kidokoro MRI score^b^	1.6 (2.1)	4.5 (4.9)	**0.01**
Sex (male)	37/70 (53%)	14/23 (60%)	0.50
Extremely preterm (<28 weeks)	21/70 (30%)	11/23 (48%)	0.12
Postnatal steroids	3/70 (4%)	7/23 (30%)	**0.006**
Surgery	4/70 (6%)	4/23 (17%)	0.08
Bronchopulmonary dysplasia	13/70 (19%)	13/23 (56%)	**0.006**
Retinopathy of prematurity	0/70 (100%)	4/23 (17%)	**0.005**

^a^Bold represents statistical significance (*P*<0.05)

^b^Data are presented as means (standard deviations). *P*-values are Bonferroni adjusted

Compared with the normal outcome group, the impaired group had a lower gestational age at birth (28.1 weeks vs. 29.3 weeks, *P=*0.03) and a lower birth weight (1094 g vs. 1314 g, *P=*0.02). Furthermore, the impaired group required longer ventilation (13.4 vs. 2.3 days, *P*<0.001), had lower 2nd Apgar scores (7.9 vs. 8.7, *P=*0.01), and had higher Kidokoro MRI abnormality scores (4.5 vs. 1.6, *P=*0.01). The incidences of postnatal steroid use (30% vs. 4%, *P<*0.001), bronchopulmonary dysplasia (56% vs. 19%, *P<*0.001), and retinopathy of prematurity (17% vs. 0%, *P<*0.001) were significantly higher in the impaired development group compared to the normal outcome group. The skull circumference at birth was lower in the impaired outcome group (25.9 cm vs. 27.4 cm) than in the impaired development group, but this difference was only borderline significant (*P=*0.05) in this cohort.

### Synthetic magnetic resonance imaging measurements and postmenstrual age at time of scan

We found significant correlations between postmenstrual age at time of scan and several MRI measurements. Specifically, the T1 relaxation time in the posterior limb of the internal capsule showed a correlation coefficient (*r*) of 0.38 with a significant negative slope of -51.3 ms/week (*P<*0.001), indicating shorter relaxation times with increasing age. Similarly, T2 relaxation time in the same region had a correlation coefficient of 0.50 and a negative slope of -5.0 ms/week (*P<*0.001). Intracranial volume and brain parenchymal volume also correlated with postmenstrual age at scan, with correlation coefficients of 0.24 (*P=*0.02; slope, 19.64 ml/week) and 0.39 (*P<*0.001; slope, 24.11 ml/week), respectively. These values were adjusted using the formula: Corrected_Value = Measured_Value + Slope × (40 - Postmenstrual_Age). All subsequent analyses utilized these adjusted values.

### Synthetic magnetic resonance imaging measurements and Bayley-III motor/cognitive outcomes at 2 years of age

The Bayley-III cognition scores showed the strongest correlations with T1 relaxation time in the posterior limb of the internal capsule (*r*=-0.37, *P<*0.0001) and with brain parenchymal volume (*r*=0.35, *P=*0.001). Weak correlations were found with the intracranial volume (*r*=0.23, *P=*0.03), T2 relaxation time in the posterior limb of the internal capsule (*r*=-0.25, *P=*0.02), and central white matter (*r*=-0.22, *P=*0.04).

Similar findings were observed for Bayley-III motor scores, with the strongest associations found with T1 relaxation time in the posterior limb of the internal capsule (*r*=-0.39, *P<*0.0001) and with brain parenchymal volume (*r*=0.41, *P<*0.0001). Weak correlations were also found between the T2 relaxation time in the posterior limb of the internal capsule (*r*=-0.28, *P=*0.008), T1 and T2 relaxation times in the central white matter (*r*=-0.22, *r*=-0.24; *P<*0.05), and intracranial volume (*r*=0.33; *P<*0.001).

After applying the Bonferroni correction for multiple comparisons, with a significance threshold of *P<*0.004, only the correlations involving T1 relaxation time in the posterior limb of the internal capsule and brain parenchymal volume for both cognition and motor scores remained statistically significant.

Of note, a significant correlation was also observed between the cognition scores and motor scores (*r*=0.65, *P<*0.0001).

No significant relationship was observed between the Bayley-III cognitive and motor outcome scores and the relaxation times of the frontal or parietal white matter, or with the cerebrospinal fluid or brain parenchymal fraction, defined as the proportion of the brain parenchymal volume to the intracranial volume.

### Synthetic magnetic resonance imaging parameters between normal outcome and impaired development groups

**Fig. 3:**
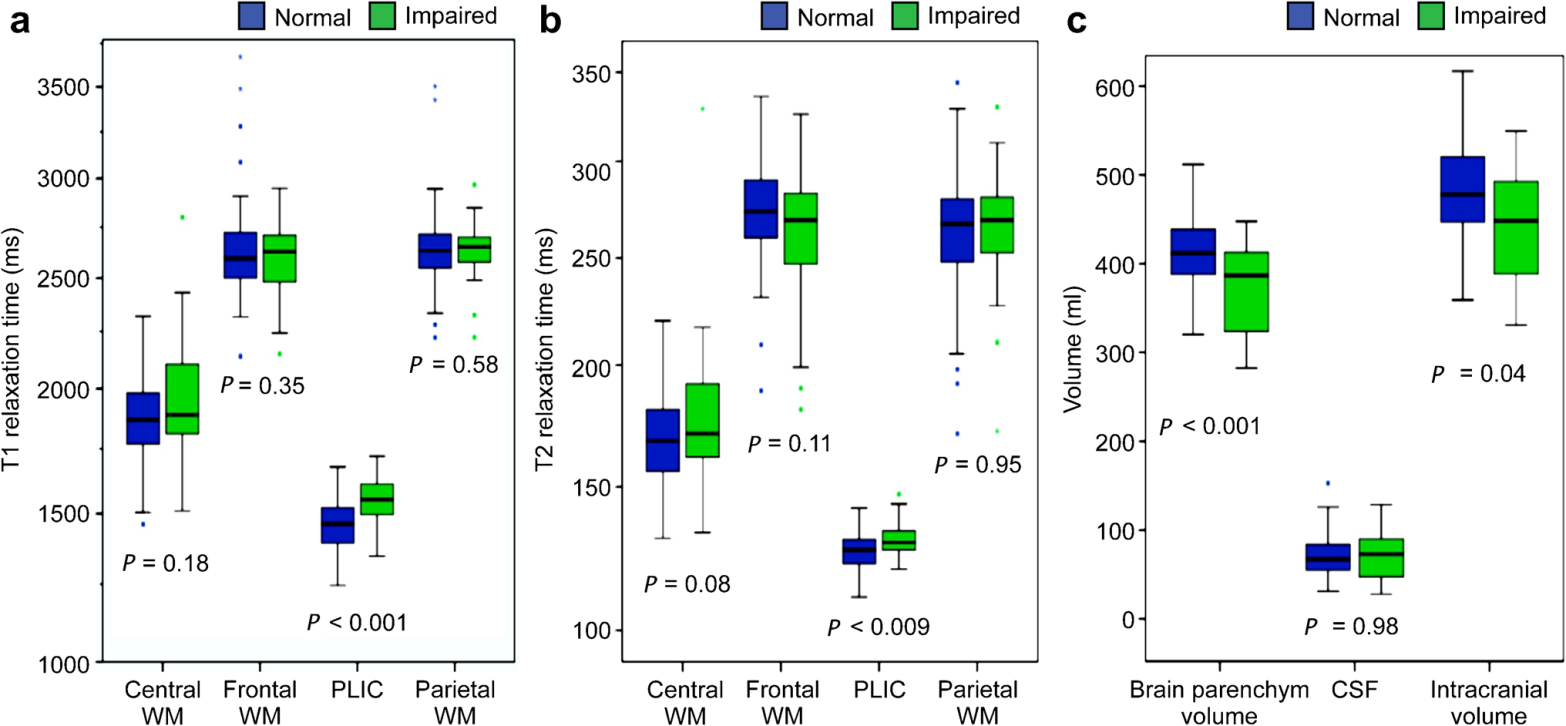
Boxplots of differences in synthetic magnetic resonance parameters between normal and impaired development groups for T1 relaxation time (**a**), T2 relaxation time (**b**), and volume (**c**). Notably, only the T1 relaxation times in the posterior limb of the internal capsule and brain parenchymal volume displayed significant differences (significance threshold =*P*<0.004; Bonferroni corrected) between the two groups. *CSF* cerebrospinal fluid, *PLIC* posterior limb of the internal capsule, *WM* white matter

The T1 relaxation time in the posterior limb of the internal capsule region was significantly prolonged in infants with impaired development compared to those with normal development, with an increase of 6% (*P<*0.001). However, after applying the Bonferroni correction for multiple comparisons, we found no significant differences in the relaxation times of other brain regions such as the frontal, parietal, or central white matter (Fig. 3).

Additionally, brain parenchymal volume was significantly reduced in infants with impaired development compared to those with normal development, with an average reduction of 10% (*P<*0.001). However, we found no notable differences in the cerebrospinal fluid volume or brain parenchymal fraction between the two groups.

### Receiver operating characteristics analysis for prediction of impaired development

**Fig. 4:**
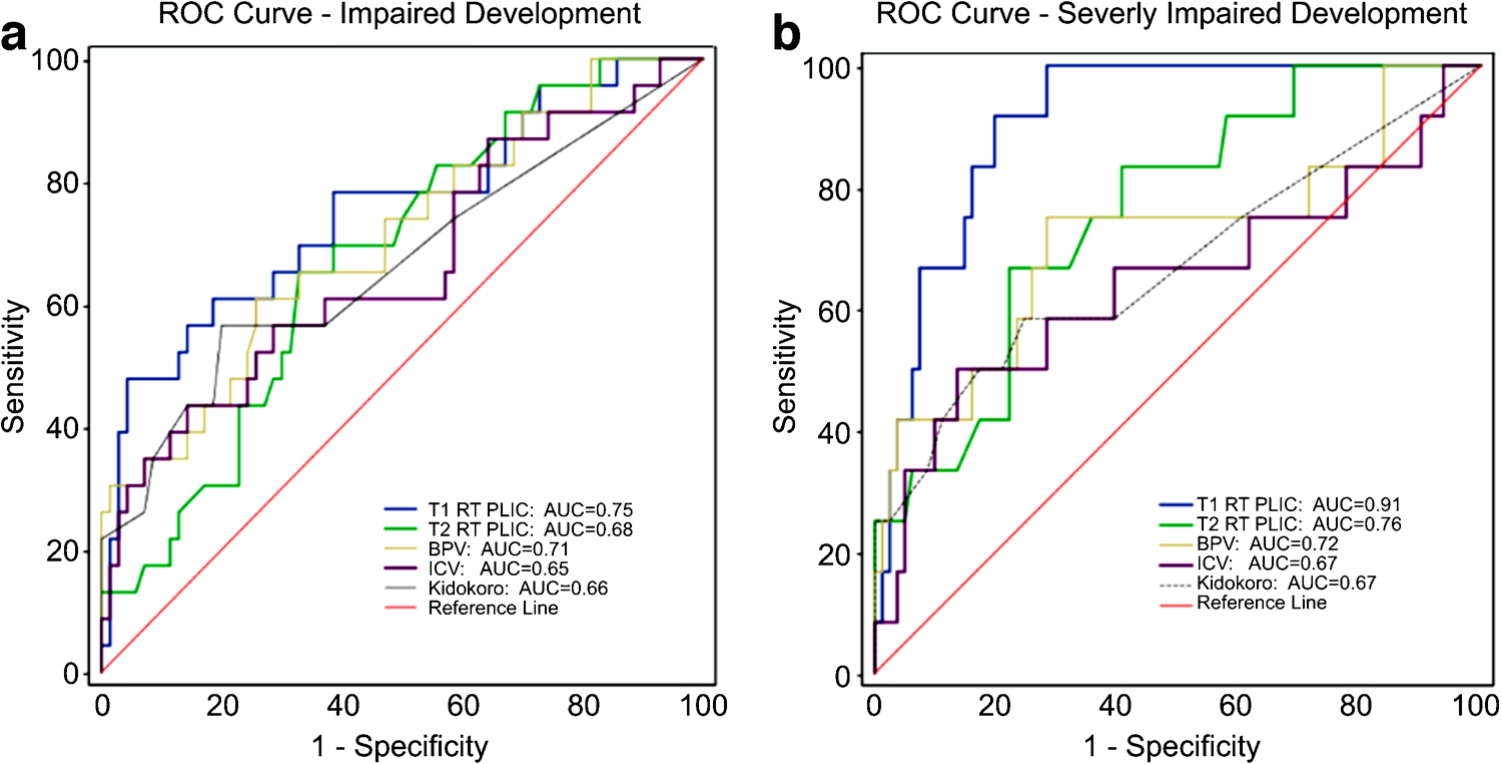
Receiver operator characteristic (ROC) curves to predict “impaired development” (**a**) and “severely impaired development” (**b**) using synthetic magnetic resonance imaging measurements. *AUC* area under the curve, *BPV* brain parenchymal volume, *ICV* intracranial volume, *PLIC* posterior limb of the internal capsule, *RT* relaxation time

The receiver operating characteristics (ROC) curves, which are presented in Fig. 4 and further detailed in Online Supplementary Material 2, evaluate the predictive capacity of synthetic MRI measurements for developmental impairment at 2 years of age for severe impairment (Bayley-III motor or cognition score <70). The T1 relaxation time in the posterior limb of the internal capsule was the most potent predictor, achieving an outstanding AUC of 0.91 [30] with an optimized Youden’s index cutoff value of 1534 ms, yielding a sensitivity of 92% and a specificity of 80% [31]. The T2 relaxation time in the posterior limb of the internal capsule showed a significant predictive value with an AUC of 0.76 and an optimized cutoff value of 131 ms, resulting in a sensitivity of 67% and a specificity of 78%. Other significant predictors included brain parenchymal volume (BPV; AUC=0.72) with a cutoff of 389 ml, which resulted in a sensitivity of 75% and a specificity of 72%, and intracranial volume (ICV; AUC=0.64) with a cutoff of 424 ml, yielding a sensitivity of 50% and a specificity of 85% (*P<*0.05). The Kidokoro MRI score showed a trend towards predictive value for severe impairment (AUC=0.67, *P=*0.06), although it did not reach statistical significance.

When the analysis was broadened to include children with mild or severe impairment (Bayley-III motor or cognition score <80), the AUC values ranged from 0.66 to 0.75, indicating moderate predictive capacity (*P<*0.05). Notably, the Kidokoro MRI score demonstrated statistical significance for impaired development in this group, with an AUC of 0.66 (*P<*0.05). Using stepwise logistic regression for the relaxation times of the posterior limb of the internal capsule, brain parenchymal volume, and intracranial volume, only the T1 relaxation time of the posterior limb of the internal capsule was maintained, which did not increase the area under the curve.

## Discussion

Our study identified significant associations in very preterm (<32 weeks) neonates at term-equivalent age between longitudinal (T1) relaxation times, as well as brain parenchymal volume, and motor and cognitive scores at 2 years.

Brain volume measurements in neonates have traditionally been limited to specialized research centers owing to the complexity of the post-processing pipelines needed for this task; however, synthetic MRI now enables fully automated neonatal brain volume measurements in a clinical setting. In line with previous studies that utilized advanced segmentation techniques [11, 12, 32, 33], we found that brain parenchymal and intracranial volumes were significantly lower in infants with impaired development. For the first time, we have been able to propose cutoff values that may guide daily decision-making [34].

During brain development, microstructural changes in water content and compartmentalization largely influence the T1 and T2 relaxation times, reflecting processes of maturation and myelination [19]. Relaxation times provide valuable insights into brain maturation, superior to visual evaluation. T1 relaxation times are particularly sensitive to fat and myelin content, whereas T2 relaxation times primarily respond to water content, reflecting different aspects of neural tissue composition and development [14, 35, 36]. Nevertheless, conventional methods of measuring relaxation times are time-consuming, and as a consequence, they have not been integrated into standard daily practices. Two recent feasibility studies demonstrated the potential predictive value of rapid synthetic MRI-based relaxometry in clinical settings, focusing on small, highly selected groups of high-risk preterm and near-term (<37 weeks) neonates with ischemia or seizures [27, 28]. However, to the best of our knowledge, no studies have yet defined the relationship between synthetic MRI-based relaxation and volumetry at term-equivalent age and outcomes in a large, non-selected cohort of high- and low-risk very-preterm (<32 weeks) neonates.

We found a strong association with T1 relaxation times and neurocognitive outcome scores in the posterior limb of the internal capsule. The posterior limb of the internal capsule, as a part of the corticospinal tract, is the most myelinated cerebral white matter structure at term-equivalent age. Aligning with previous research, delayed myelination in the posterior limb of the internal capsule is linked to impaired long-term cognitive and motor outcomes [37]. Although the posterior limb of the internal capsule is mainly a motor pathway, our results support the notion that decreased myelination in the posterior limb of the internal capsule is closely associated with diffuse axonal disease and overall brain development [38, 39]. These findings align with the results of a previous study in the same cohort, where we reported prolonged relaxation times in the posterior limb of the internal capsule in neonates with severe postnatal morbidity.

We were unable to confirm the findings of Kim et al. [27], who demonstrated prolonged relaxation times in the frontal and parietal white matter in preterm infants with later adverse outcomes. This discrepancy might arise from the differences in the selection of ROIs or, more importantly, the patient populations studied; unlike our non-selected very preterm infant cohort, the study by Kim et al. also included near-term (<37 weeks) infants with acute hypoxia or seizures, conditions that may cause edema and prominent white matter signal alterations. Additionally, high T2 signal areas are commonly observed in the frontal and parietal white matter at term-equivalent age, and our study supports the conclusion of a recent meta-analysis, which indicates that these areas of diffuse excessive high signal intensities are not predictive of neurocognitive outcomes or cerebral palsy [40].

In contrast to the posterior limb of the internal capsule, the frontal and parietal white matter exhibit minimal myelination at term-equivalent age. It is widely accepted that white matter structures undergoing early myelination are more vulnerable to injury related to prematurity. Alternatively, this might indicate that injuries to myelin-forming oligodendrocytes only become evident during periods of active myelination [41].

In this study, we also propose cutoff values for relaxometry and brain parenchymal volume measurements as predictors of developmental impairment.

With a sensitivity of 92% and a specificity of 80%, a cutoff value of 1534 ms for the T1 relaxation time in the posterior limb of the internal capsule proved to be a robust and reliable indicator of severely impaired development (AUC = 0.91). Furthermore, T2 relaxation times longer than 131 ms in the posterior limb of the internal capsule and brain parenchymal volume less than 389 ml were also significant predictors of severe impairment (AUC, 0.76 and 0.71, respectively). When we broadened our analysis and included infants with mild impaired development, using the same cutoff values, the T1 relaxation time in the posterior limb of the internal capsule and brain parenchymal volume remained significant predictors of impaired motor or cognitive outcomes. Notably, the predictive power of the T1 and T2 relaxation times in the posterior limb of the internal capsule (AUC, 0.75 and 0.68, respectively) and brain parenchymal volume (AUC, 0.71) surpassed the diagnostic performance of the widely used qualitative Kidokoro MRI scores (AUC 0.66), which utilize simple 2-dimensional linear measurements and structural assessments to evaluate brain volume and focal parenchymal lesions in premature infants at term-equivalent age [30].

Our study has several limitations. All examinations were performed using the same 3T MRI scanner, which may have limited the generalizability of our findings. It is possible that the quantitative values of other MRI scanners are different and that the proposed cutoff values could differ. However, previous studies have shown that synthetic MRI-based relaxation time and volume measurements of different scanners yield robust results [42, 43]. We could not compare our findings with those of healthy full-term born controls, and it would have been insightful to conduct serial imaging to better understand the changes in brain maturation among preterm infants with and without impaired development. Future research should aim to validate these findings using different MRI scanners and investigate whether a combination of qualitative and quantitative MRI parameters offers superior performance in predicting neurodevelopmental outcomes.

In conclusion, synthetic MRI translates neonatal brain volume measurements and relaxometry from research into daily clinical practice. We found a robust association between relaxation times in the posterior limb of the internal capsule, global brain volume, and neurocognitive outcomes at 2 years of age. These results underscore the potential of synthetic MRI relaxometry and volumetry in identifying preterm infants who could benefit from early neurorehabilitation interventions, ultimately improving the quality of life for these at-risk children.

### Supplementary Information

Below is the link to the electronic supplementary material.

Supplementary file1 (DOCX 21 KB)

Supplementary file2 (DOCX 22 KB)

## Data Availability

The data supporting the findings of this study will be made available upon reasonable request to the corresponding author.
