# Supplementary material for: Synthetic magnetic resonance-based relaxometry and brain volume: cutoff values for predicting neurocognitive outcomes in very preterm infants
Source: Pediatr Radiol. 2024 Jul 9;54(9):1523–31. doi: 10.1007/s00247-024-05981-x (PMC11324712; doi:10.1007/s00247-024-05981-x)
Supplement: Supplementary file 1 — Supplementary file1 (DOCX 21 KB) [file 247_2024_5981_MOESM1_ESM.docx]

**Supplementary Material 1 – Magnetic Resonance Imaging Acquisition parameters (Phlips Ingenia 3 Tesla)**

|  | Synthetic MRI | Sag 3-D T2WI | Sag 3-D T1WI | SWI | DWI | Multishell DTI |
| --- | --- | --- | --- | --- | --- | --- |
| FoV (mm) | 181 × 150 × 105 | 160 × 160 × 180 | 175 × 175 × 110 | 150 × 124 × 100 | 180 × 180 × 139 | 200 × 200 × 100 |
| Voxel size (mm) | 0.7 × 0.9 × 3.0 | 0.9 × 0.9 × 0.9 | 1.0 × 1.0 × 1.0 | 0.6 × 0.6 × 2.0 | 1.5 × 1.9 × 4 | 2.0 × 2.0 × 2.0 |
| Slice gap (mm) | 0.3 | - | - | - | 1 | 0 |
| TR (ms) | 5000 | 2500 | 6.076 | 31 | 3870 | 6766 |
| TE (ms) | 13 / 100 | 331 | 2.729 | 7.2 | 103 | 106 |
| TI (ms) | 4 TI's. default | - | 800 | - | - | 220 |
| ETL (ms) | 10 | 117 | 141 | - | - | - |
| SENSE factor | 1.7 | 2 / 2.5 | 1 | 2 / 1 | 2 | 2.2 |
| Bandwitdh (kHz) | 220.5 | 457 | 298 | 254 | 1436 | 1913 |
| Averages | 1 | 1 | 1 | 1 | 1 | 1 |
| Flip angle (degrees) | 90 | 90 | 8 | 17 | 90 | 90 |
| Scan time (min:sec) | 6:10 | 2:00 | 3:37 | 2:42 | 0:46 | 16:34 |

*DTI* diffusion tensor imaging, *DWI* diffusion weighted imaging, *MRI* magnetic resonance imaging, *SWI* susceptibility weighted imaging
