# Supplementary material for: Synthetic magnetic resonance-based relaxometry and brain volume: cutoff values for predicting neurocognitive outcomes in very preterm infants
Source: Pediatr Radiol. 2024 Jul 9;54(9):1523–31. doi: 10.1007/s00247-024-05981-x (PMC11324712; doi:10.1007/s00247-024-05981-x)
Supplement: Supplementary file 2 — Supplementary file2 (DOCX 22 KB) [file 247_2024_5981_MOESM2_ESM.docx]

**Supplementary Material 2. Area under the receiver operating characteristics (ROC) curve**

|  | AUC | 95% CI | Cut-off | Sensitivity | Specificity | *P* |
| --- | --- | --- | --- | --- | --- | --- |
| Impaired (*n=*23/93) |  |  |  |  |  |  |
| T1 relaxation time PLIC | 0.75 | 0.63–.088 | 1,534 ms | 61% | 81% | <0.001 |
| T2 relaxation time PLIC | 0.68 | 0.56–.079 | 131 ms | 43% | 77% | 0.01 |
| Brain parenchymal volume | 0.71 | 0.58–0.83 | 389 mL | 61% | 74% | 0.03 |
| Intracranial volume | 0.66 | 0.52–0.79 | 424 mL | 39% | 87% | 0.02 |
| Kidokoro MRI score | 0.66 | 0.52–0.81 | 2 | 57% | 80% | 0.02 |
| Severely impaired (*n=*11/93) |  |  |  |  |  |  |
| T1 relaxation time PLIC | 0.91 | 0.84–0.97 | 1,534 ms | 92% | 80% | <0.001 |
| T2 relaxation time PLIC | 0.76 | 0.62–0.90 | 131 ms | 67% | 78% | 0.004 |
| Brain parenchyma volume | 0.72 | 0.53–0.90 | 389 mL | 75% | 72% | 0.02 |
| Intracranial volume | 0.64 | 0.44–0.83 | 424 mL | 50% | 85% | 0.11 |
| Kidokoro MRI score | 0.67 | 0.48–0.86 | 2 | 58% | 75% | 0.06 |

AUC analysis is used to distinguish patients with impaired (Bayley-III <80) and severely impaired (Bayley-III <70) development from infants with normal development based on synthetic MRI-derived relaxation time, brain parenchymal and intracranial volume measurements, and the Kidokoro MRI score.
*P<*0.05 is defined as significant*. AUC* area under the curve, *CI* confidence interval, *MRI* magnetic resonance imaging, *PLIC* posterior limb of the internal capsule, *ROC* receiver operating characteristic
